# Comparative genomics of *Borrelia lusitaniae*

**DOI:** 10.1093/g3journal/jkaf319

**Published:** 2026-01-12

**Authors:** Isabel Lopes de Carvalho, Maria Sofia Núncio, Ana C Norte, Emmanuel F Mongodin, Benjamin J Luft, Steven E Schutzer, Xiaohua Yang, Claire M Fraser, Sabrina Hepner, Volker Fingerle, Gabriele Margos, Rick Morgan, Saymon Akther, Weigang Qiu, Sherwood R Casjens

**Affiliations:** National Institute of Health, Dr. Ricardo Jorge Centre for Vector and Infectious Diseases Research, Águas de Moura, Setúbal 2965-575, Portugal; National Institute of Health, Dr. Ricardo Jorge Centre for Vector and Infectious Diseases Research, Águas de Moura, Setúbal 2965-575, Portugal; Department of Life Sciences, University of Coimbra, MARE-Marine and Environmental Sciences Centre, Coimbra, Coimbra 3004-456, Portugal; Institute for Genome Sciences, University of Maryland School of Medicine, Baltimore, MD 21021, United States; Department of Medicine, Renaissance School of Medicine, Stony Brook University (SUNY), Stony Brook, NY 11794, United States; Department of Medicine, Rutgers New Jersey Medical School, Newark, NJ 07103, United States; Department of Medicine, Renaissance School of Medicine, Stony Brook University (SUNY), Stony Brook, NY 11794, United States; Institute for Genome Sciences, University of Maryland School of Medicine, Baltimore, MD 21021, United States; Bavarian Health and Food Safety Authority and German National Reference Centre for Borrelia, Oberschleissheim, 2, Bavaria 85764, Germany; Bavarian Health and Food Safety Authority and German National Reference Centre for Borrelia, Oberschleissheim, 2, Bavaria 85764, Germany; Bavarian Health and Food Safety Authority and German National Reference Centre for Borrelia, Oberschleissheim, 2, Bavaria 85764, Germany; New England BioLabs, Ipswich, MA 01938, United States; Hunter College, City University of New York, New York, NY 10021, United States; Hunter College, City University of New York, New York, NY 10021, United States; Department of Systems and Computational Biomedicine, Weill Cornell Medical College, New York, NY 10021, United States; Pathology Department, University of Utah School of Medicine and School of Biological Sciences, Salt Lake City, UT 84112, United States

**Keywords:** *Borrelia lusitaniae*, Lyme disease, whole genome sequence, linear plasmid, OspC protein, genome assembly

## Abstract

Human Lyme disease is a frequent tick-borne human disease that is caused by several species in the *Borrelia burgdorferi* sensu lato (BBSL) clade of the bacterial spirochete genus *Borrelia*. Although *Borrelia lusitaniae* does not appear to be a major cause of this disease, it has been isolated from human patients. This *Borrelia* species is unusual in that its vertebrate reservoir includes reptiles in Europe and North Africa. We describe here the complete genome sequences of three *B. lusitaniae* isolates, PotiB2^T^ (the species type strain) and PotiB3 that represent a Southern Portugal-North African lineage and PoHL1 that represents a Northern Portugal-Central Europe lineage. Like other members of this genus, their genomes include linear chromosomes as well as numerous circular and linear plasmids. Their total genomes contain 1,202,579 bp (PotiB2^T^), 1,171,499 bp (PotiB3), 1,155,617 bp (PoHL1), and they carry eight (PotiB2^T^ and PotiB3) or six (PoHL1) plasmids. We discuss the differences and similarities of these genomes with other members of the BBSL species group. A most unusual feature of the *B. lusitaniae* genomes is that their important cp26 plasmids are partially degraded dimers of the cp26 plasmid that is present in all other BBSL isolates analyzed to date. The cp26 plasmid has been shown to be essential for growth of *B. burgdorferi* sensu stricto B31^T^. The *B. lusitaniae* dimer cp26 plasmids carry multiple *ospC* genes of different types, which is unique to this species. OspC is an important protein that is required for the establishment of mammalian infection by *B. burgdorferi* B31^T^ and tick salivary gland infection in *Borrelia afzelii*. It remains unclear how genes of more than one OspC type in one strain might affect the infection process.

## Graphical Abstract

**Graphical Abstract:**
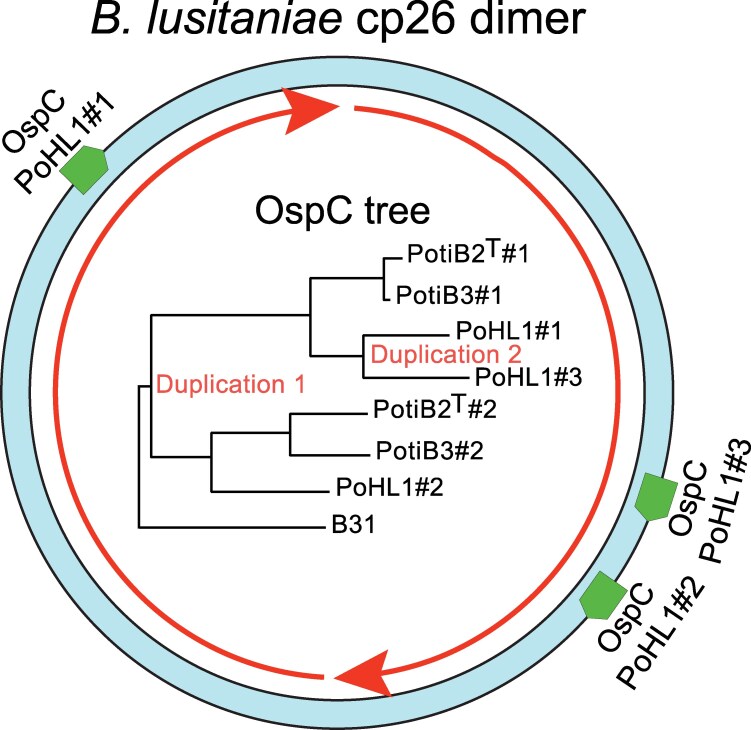

## Introduction

Human Lyme disease is a frequent tick-borne human disease that is caused by several members of a clade of related bacterial species in the genus *Borrelia*. This clade, which currently contains 23 species, is called *Borrelia burgdorferi* sensu lato (BBSL) or the Lyme agent *Borrelias*. Four species in this group have been reported to cause most cases of human disease, *B. burgdorferi* (sensu stricto) in North America and Europe, and *Borrelia afzelii, Borrelia bavariensis*, and *Borrelia garinii* in Eurasia. In addition, *Borrelia spielmanii* and *Borrelia mayonii* are human pathogens, while *Borrelia bissettiae* and *Borrelia lusitaniae* have been reported to infect humans in a few cases (Collares-Pereira et al. 2004; da Franca et al. 2005; Vitorino et al. 2008; de Carvalho et al. 2008a; Margos et al. 2010; Stanek and Reiter 2011; Coipan et al. 2016; Kingry et al. 2016; Radolf et al. 2021). Whole genome sequences for seven of the eight human infecting species have been reported (Fraser et al. 1997; Glöckner et al. 2004; Casjens et al. 2011; Schutzer et al. 2012; Becker et al. 2020; Margos et al. 2023), and we recently reported partial genome sequences of three *B. lusitaniae* isolates (the missing eighth species) but have not described them in detail (Akther et al. 2024). Here, we describe complete genome sequences of these three isolates which include the sequences of 13 plasmids whose sequences have not been previously reported.

*Borrelia lusitaniae* was first isolated in 1993 from a tick in Portugal (Núncio et al. 1993), and the species was formally described in 1997 (Le Fleche et al. 1997). Since then it has been found elsewhere in central and southern Europe as well as North Africa (Zhioua et al. 1999; Sarih et al. 2003; Wodecka and Skotarczak 2005; Bertolotti et al. 2006; Dsouli et al. 2006; Majlathova et al. 2006; de Carvalho et al. 2008b; Taragelova et al. 2016; Cakic et al. 2019; Okeyo et al. 2020; Del Cerro et al. 2022; Musilova et al. 2022). In Portugal, for example, it can be the most prevalent *Borrelia* species in questing ticks (De Michelis et al. 2000; Baptista et al. 2004; de Carvalho et al. 2008b; Taragelova et al. 2016; Estrada-Pena et al. 2018). *B. lusitaniae* is commonly found in *Ixodes ricinus* tick adults, larvae and nymphs feeding on Algerian *Psammodromus* lizards, *Podarcis muralis* and *Teira dugesii* wall lizards, and *Lacerta viridis* green lizards, as well as on small mammals; however, it is found only rarely in tick adults, larvae or nymphs feeding on birds (Baptista et al. 2004; Dsouli et al. 2006; Majlathova et al. 2006; Poupon et al. 2006; Richter and Matuschka 2006; Amore et al. 2007; de Carvalho et al. 2008b, 2010; Ragagli et al. 2011; De Sousa et al. 2012; Norte et al. 2013; Norte et al. 2015; Tomassone et al. 2017; Sukara et al. 2018; Musilova et al. 2022). *B. lusitaniae* has also been identified in green lizards (*L. viridis*), sand lizards (*Lacerta agilis*), common wall lizards (*P. muralis*), and Balkan wall lizards (*Podarcis taurica*) (Bertolotti et al. 2006; Amore et al. 2007; Foldvari et al. 2009; Ekner et al. 2011; Musilova et al. 2022) as well as small mammals in Germany (Krol et al. 2022) and Portugal (de Carvalho et al. 2010), suggesting that these likely comprise parts of the vertebrate reservoirs in these regions.

Since *B. lusitaniae* has been found to infect humans and is a potential human pathogen (Collares-Pereira et al. 2004; da Franca et al. 2005; Vitorino et al. 2008; de Carvalho et al. 2008a; Veinovic et al. 2025), it is important to develop improved detection, diagnosis, prevention and treatment methods for this bacterial species. Here, we analyze and compare whole genome sequences of the *B. lusitaniae* type strain PotiB2^T^ (Núncio et al. 1993; Le Fleche et al. 1997) and two other isolates, PotiB3 (Núncio et al. 1993) and PoHL1 (Collares-Pereira et al. 2004). Multilocus sequence typing (MLST) analysis has indicated that there are two major extant clades within the *B. lusitaniae* species (Grego et al. 2007; Vitorino et al. 2008; Norte et al. 2021; Cirkovic et al. 2024). PotiB2^T^ and PotiB3 represent a Southern Portugal-Mediterranean-North African lineage and PoHL1 represents a Northern Portugal-Central Europe lineage.

## Methods

### Strain sources, growth and DNA isolation

Strains PotiB2^T^ (Núncio et al. 1993; Le Fleche et al. 1997), PotiB3 (Núncio et al. 1993) and PoHL1 (Collares-Pereira et al. 2004) were from our strain collections. Bacteria were grown in in-house-made MKP medium using standard procedures (Preac-Mursic et al. 1986; Hepner et al. 2023). Cultures were grown to a density of 1 × 10^8^ cells per mL and genomic DNA was extracted at the German National Reference Centre for Borrelia, Oberschleissheim, using the Maxwell 16 LED DNA kit (Promega, Germany) according to the manufacturer's protocol.

### Whole genome sequencing, assembly and analysis

Methods used in the determination of the genome sequences for *B. lusitaniae* strains PotiB2^T^, PotiB3 and PoHL1 are described in Akther et al. (2024). The PotiB2^T^ genome sequence was determined at the Institute for Genome Sciences, University of Maryland School of Medicine, Baltimore, MD, and those of PotiB3 and PoHL1 were determined at New England BioLabs, Ipswich, MA.

The sequences were manually curated as follows: Pacific Biosciences SMRT (Single Molecule Real-Time) sequencing runs proceed around the covalently closed hairpin termini to generate long (up to 8–10 kbp) inverted repeat “wrapround” sequences. Sequence could therefore be determined to the tips of most of the linear replicons. When present, the “outside” halves of terminal “wraparound” inverted repeats with *Borrelia* telomere consensus motifs near their centers were manually trimmed from the ends of linear contigs. Direct terminal repeats were merged to circularize plasmid contigs where appropriate. Genome annotation was performed using the NCBI prokaryotic genome annotation pipeline (Tatusova et al. 2016; Li et al. 2021) through the NCBI genome submission portal (https://submit.ncbi.nlm.nih.gov). Sequences are available in NCBI BioProject PRJNA431102 and GenBank accession numbers of the individual replicons are listed in Supplementary Table 1.

Chromosomal gene content analysis was performed by careful manual comparison using the chromosome synteny browser at the BorreliaBase web site (borreliabase.org) (Di et al. 2014), followed by more detailed comparisons of regions of difference using BLASTp, BLASTn, tBLASTn (Altschul et al. 1997), and DNA Strider (Douglas 1994). The neighbor-joining tree in Supplementary Fig. 1 was constructed with Clustal X (Larkin et al. 2007).

## Results and discussion

### Borrelia lusitaniae main chromosomes

The whole genomes of *B. lusitaniae* isolates PotiB2^T^ (the type strain for this species), PotiB3 and PoHL1 were sequenced as reported in Akther et al. (2024) and in the Methods section of this report. Since SMRT sequencing runs read around the covalently closed hairpin termini, sequence was determined to the tips of nearly all their linear replicons (see Telomeres section below). The linear chromosomes of these three isolates are 903,614 bp, 903,383 bp, and 903,092 bp long, respectively, and are largely syntenic with those of other BBSL species. They form a deep branch within the “Eurasian” clade of BBSL species (see Fig. 4 in Akther et al. (2024) and Fig. 1 in Becker et al. (2016)). The sequence data from these three isolates also assembled into a number of plasmids that increase their total genome sequence sizes to 1,202,579 bp, 1,171,499 bp, and 1,115,561 bp, respectively. Their replicons are listed in Table 1 (most of the plasmids have not been previously published).

**Table 1.** *Borrelia lusitaniae* replicons.

	Length (bp)			
Replicon	PotiB2^T^	PotiB3	PoHL1	Comments
Chromosome	903,614	903,383	903,092	Linear
Cp26	45,668	44,880	49,559	Unique circular dimer; multiple *ospC* genes
Cp32-1	–	28,974	–	Circular; monomer cp32-1
Cp32-12	–	28,947	–	Circular; monomer cp32-12
Cp32-12 + 28-4	58,174	–	–	Circular dimer; cp32-12 and lp28-4 PFam32 genes
Cp32-28-4	–	29,232	28,850	Circular; monomer cp32-28-4 PFam32 gene
Lp17	12,546	15,446	16,582	Linear; PFam44 genes
Lp25	21,336^a^	30,664	24,822	Linear; *pncA* and *bptA* genes
Lp28-8	27,662	26,187	27,592^a^	Linear; *vls*/*vlsE* and *sagABCDEF* toxin genes
Lp56 + 32-3	42,115	–	–	Linear; four PFam32 genes (see text)
Lp38	27,543	–	–	Linear; cp32-like *erp* genes
Lp54	63,921	63,786	65,054	Linear; left-end PFam60 genes
Linear plasmid total	195,123	136,083	134,060	
Circular plasmid total	103,843	132,033	78,409	
Genome total	1,202,579	1,171,499	1,115,561	

^a^Sequence does not extend to the tips of at least one of the telomeres.

The PotiB2^T^ and PotiB3 chromosomes are 99.7% identical to one another, while the PoHL1 chromosome is 98.6% identical to both PotiB2^T^ and PotiB3 (calculated by Nucmer; Marcais et al. 2018). The *B. lusitaniae* genomes are rather distantly related to other BBSL species; for example, PotiB2^T^ chromosome is 7.2, 7.6, 7.9, and 8.8% different (by Nucmer) from those of *B. afzelii* PKo, *Borrelia japonica* HO14, *B. burgdorferi* B31^T^ and *Borrelia sinica* CMN3, respectively. These relationships are in agreement with previous multilocus sequence type (MLST) analyses (Grego et al. 2007; Vitorino et al. 2008; Norte et al. 2021) which showed that *B. lusitaniae* chromosomes form two groups—one that includes isolates from southern Portugal and North Africa that includes PotiB2^T^ and PotiB3, and one from northern Portugal and central Europe that includes PoHL1.

The tip regions of BBSL chromosomes are particularly variable, and often some have several kbp of terminal plasmid-like sequences that extend the linear chromosomes relative to the minimal chromosome “constant region” (Huang et al. 2004; Casjens et al. 2017; Casjens et al. 2018; Margos et al. 2019; Akther et al. 2024). The PotiB2^T^, PotiB3 and PoHL1 chromosomes have only 962, 635 and 771 “extra” bp to the left of the leftmost gene (homologs of *B. burgdorferi* sensu stricto strain B31^T^ gene *bb_001*) and 105, 82 and 87 “extra” bp to the right of their rightmost gene (homologs of B31^T^ gene *bb_843*), respectively (see Supplementary Fig. 1 of Akther et al. 2024). These terminal sequences contain no recognizable genes.

**Fig. 1.**
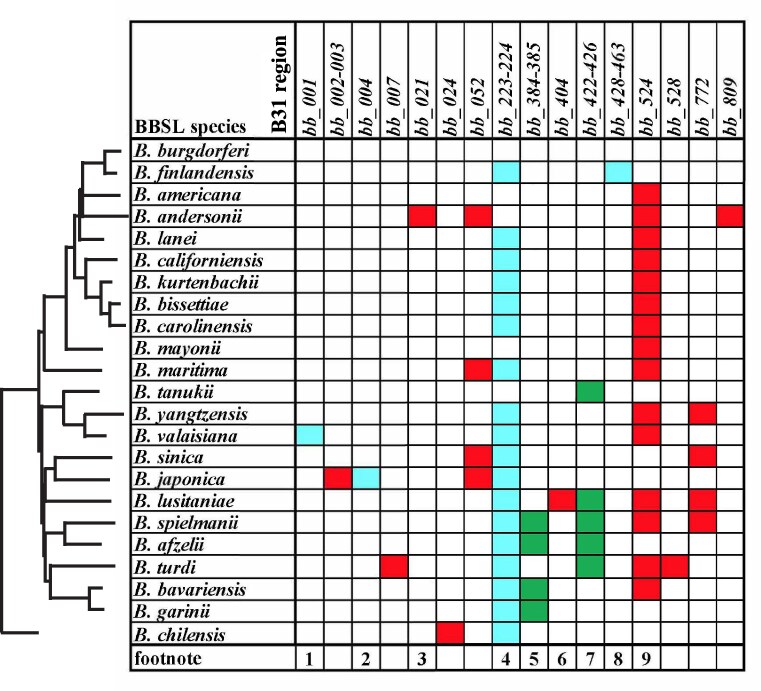
Major chromosomal protein-coding gene content differences among BBSL species. Gene content of each isolate was compared to that of *Borrelia burgdorferi* B31^T^. Indels between genes and short in-frame indels within genes are not shown, and because sequencing errors are possible, frameshifted genes were not distinguished from intact genes. Cell colors indicate deletions (red), insertions (green) and other rearrangements such as inversions gene movements and multiple indels relative to B31^T^ (blue). A phylogenetic tree of the BBSL species' chromosomes from Akther et al. (2024) is shown on the left. Footnotes: (1) Present at left instead of right end except in Am501; (2) inverted in *B. japonica*; (3) *bb_021* is also a frameshifted or truncated pseudogene in some *B. burgdorferi* and *B. finlandensis* isolates; (4) annotated as pseudogenes in B31^T^; appear to be fragments of lp36 gene *bb_k13*-like gene; these are very variable across the BBSL species; (5) extra gene in tandem *bmp* array (previously reported for *B. afzelii* (Mongodin et al. 2013)); (6) ∼230 bp deletion that truncates the *bb_404* homolog in PotiB2; (7) duplication of the region between B31^T^ homologs *bb_422* and *bb_426* including the 16S rRNA gene; (8) The DNA between the B31^T^ homologs *bb_427* and *b_464* is inverted in the *B. finlandensis* Z11 chromosome; it is not known if this could be a sequencing error; (9) variation in this region has been pointed out before (Mongodin et al. 2013), and each species (or isolate in some cases) has different rearrangements suggesting ongoing decay.

Analysis of the gene content of the constant portions of BBSL chromosomes has shown that they are strikingly similar. We compared the chromosomes of the 23 BBSL species using the panel of 78 isolates used by Akther et al. (2024) (listed in their Supplementary Table 1a and 1b), and Fig. 1 of this report shows their gene differences. Only thirteen of the 856 currently annotated *B. burgdorferi* B31^T^ protein-coding genes in the “constant” region are not universally present in all 23 BBSL species, The three *B. lusitaniae* chromosomes have no gene content differences, and they have only five such differences from B31^T^; homologs of B31^T^  *bb_223/224* (hypothetical; most likely a single pseudogene), *bb_404* (hypothetical)*, bb_524* (encodes a putative phosphatase), and *bb_772* (*flgI*, flagellar basal body), are missing or partially missing in *B. lusitaniae*. In addition, The *B. lusitaniae* 16S rRNA gene is duplicated relative to B31^T^. Similar duplications are present in four other Eurasian clade species, *B. afzelii, B. spielmanii, Borrelia tanukii*, and *Borrelia turdi*. The other eight variably present protein-coding genes in Fig. 1 are predicted to have the following functions: *bb_004,* phosphoglucomutase; *bb_007*, hydrolase; *bb_024*, sugar dehydrogenase; *bb_384-385,* extra gene in tandem *bmp* ABC-type nucleoside transporter substrate binding gene array (Gorbacheva et al. 2000; Cuellar et al. 2020); *bb_0528*, oxidoreductase; *bb_0021*, *bb_0052* and *bb_0809*, tRNA modification enzymes; *bb_001, bb_002-3* and *bb_428-463* are gene movements or inversions. There are typically 1 to 5 gene content differences between any two BBSL species. These differences suggest that the missing genes in Fig. 1 can be dispensable in the molecular lifestyles of the different BBSL species.

### Plasmids

Analysis of *B. lusitaniae* isolate DNAs by agarose electrophoresis has shown that, like other BBSL species, they harbor a number of linear and circular plasmids (Vitorino et al. 2010; R. van Vugt, T. Vigil and S. Casjens, unpublished). The whole genome sequences of PotiB2^T^ and PotiB3 each contain eight plasmids, and that of PoHL1 contains six plasmids (Table 1). Their circular plasmids total 103,843 bp, 132,033 bp and 78,409 bp, and their linear plasmids total 195,123 bp, 136,083 bp and 134,060 bp, respectively. The plasmids make up 24.9, 22.9, and 19.0% of these genomes, respectively. BBSL plasmids have traditionally been named according the sequence type of their encoded Paralogous Protein Family 32 (PFam32) partition protein that is thought to be involved in plasmid compatibility (Casjens et al. 2000; Casjens et al. 2010; Casjens et al. 2012; Schwartz et al. 2021). The *B. lusitaniae* plasmids have thus been named in this fashion, and the PFam32 relationships are shown for PotiB2^T^ replicons in Supplementary Fig. 1. No *Borrelia* cell is known that contains two plasmids with the same PFam32 protein type. This is true for these *B. lusitaniae* isolates as well, which lends further credence to the idea that these proteins control plasmid compatibility. A majority of their plasmid sequences were not complete when the chromosomes sequences were reported (Akther et al. 2024), but all PotiB2^T^, PotiB3 and PoHL1 plasmid sequences are now available, and their accession numbers are listed in Supplementary Table 1.

#### Cp26

The circular cp26 plasmid is universally present in BBSL isolates (Tilly et al. 1997; Byram et al. 2004; Casjens et al. 2017; Casjens et al. 2018; Hepner et al. 2023; Margos et al. 2023; Akther et al. 2024), and it has been shown to be essential for growth of *B. burgdorferi* B31^T^ in culture (Jewett et al. 2007). It is highly evolutionarily conserved and has the same syntenic gene content in all previously sequenced BBSL genomes with the exception of *B. sinica* and *Borrelia andersonii* cp26s which lack intact *guaA* and *guaB* genes (Akther et al. 2024). Cp26s encode (i) enzymes and small molecule transporters that are important in nucleotide metabolism and utilization of chitobiose and glucosamine (Tilly et al. 2004; Jewett et al. 2009; Troy et al. 2016), (ii) the protelomerase/resolvase (ResT) that creates the closed hairpin telomeres during DNA replication (Ravin et al. 2000; Chaconas et al. 2001; Kobryn and Chaconas 2002), and (iii) the important outer surface protein OspC. The latter is expressed in the tick and early in mammal infection and is required for mouse infection and tick salivary gland invasion (Grimm et al. 2004; Tilly et al. 2006; Fingerle et al. 2008; Tilly et al. 2013).

**Fig. 2.**
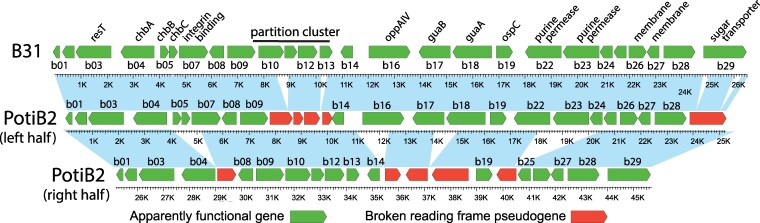
*Borrelia lusitaniae* PotiB2^T^ cp26 gene map. An open reading frame map of *B. lusitaniae* PotiB2^T^ cp26 is shown with the *Borrelia burgdorferi* B31^T^ cp26 above for comparison. The two monomer cp26 units in the dimer circle are shown on two lines. Putative genes that appear to be intact are green, and pseudogenes with broken reading frames are red. Blue shading between maps indicates convincing nucleotide similarity of the left portion of PotiB2^T^ cp26 to strain B31^T^ cp26, and between the two halves of PotiB2^T^ cp26. Black numbers below the B31^T^ plasmid genes indicate their names according to Casjens et al. (2000; 2012), and these are shown above the homologous genes in the PotiB2^T^ plasmid for clarity. Labels above genes indicate other names given to those genes. A base pair scale is shown below each map.

**Fig. 3.**
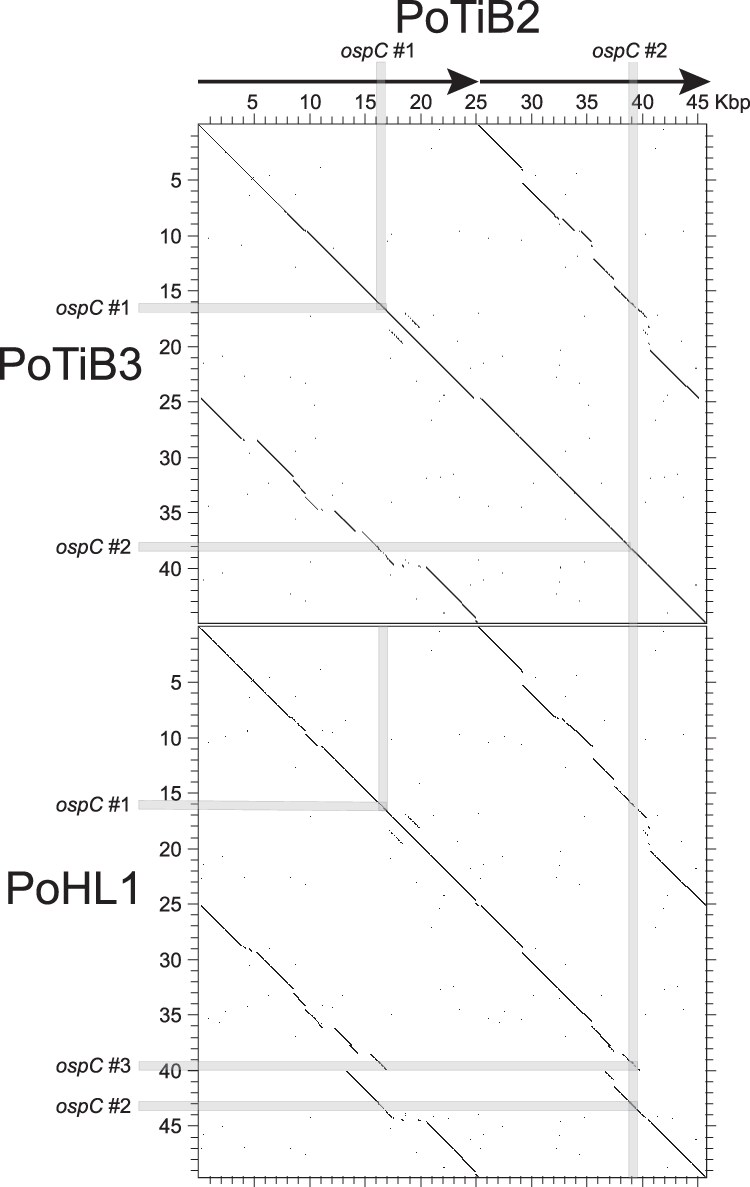
Dotplots of *Borrelia lusitaniae* cp26 dimers. The dot plot of similarly oriented cp26 sequences was constructed with DNA Strider (Douglas 1994) using a scan window stringency of 15 identities in 15 nucleotides. Strain names are shown on the left and top of the plot, and the *ospC* gene locations are indicated by gray shading.

The three *B. lusitaniae* cp26 sequences described here are structurally unique in that they are circular head-to-tail *dimers* of the canonical cp26 monomer found in all other BBSL species. Figure 2 shows an open reading frame (ORF) map of the PotiB2^T^ cp26 plasmid. This cp26 dimer must have had 26 duplicated gene pairs when it first formed (monomer cp26s have 26 genes). However, at present 14 of these pairs have one member with a broken or missing reading frame due to frameshift mutations and/or deletions, while the other member appears to be intact. This is also true for the PotiB3 and PoHL1 cp26s. Thus, unlike other BBSL cp26 plasmids, numerous pseudogenes are present in the *B. lusitaniae* cp26s. Both PotiB2^T^  *ospC* genes appear to be intact, and they are quite different from one another (see below). The PotiB3 cp26 dimer is very similar to that of PotiB2^T^, with the major difference being a 607 bp deletion at position 24,607 in the latter plasmid. The PoHL1 cp26 dimer is similar to those of the other two isolates; however, it is about 3900 bp longer than PotiB2^T^ cp26 due to the presence of a third, also apparently intact, *ospC* gene and surrounding region and a number of scattered smaller indels (see dotplot in Fig. 3). In the region with fewest indel differences, bps 1-24,730 (PotiB2^T^ coordinates), the sequence of PotiB2^T^ is 99.3% identical to PotiB3 and 97.8% identical to PoHL1 after small indels are removed. These relationships and those of the whole cp26 sequence tree shown at low resolution in Supplementary Fig. 5 of Akther et al. (2024), further support the notion of two *B. lusitaniae* clades (above). We note that complete dimers *B. burgdorferi* B31^T^ cp26 plasmids formed, presumably by homologous recombination, in experiments performed by Tilly et al. (1998). These plasmids were stably maintained, but their laboratory origins are unrelated to the dimers described here.

**Fig. 4.**
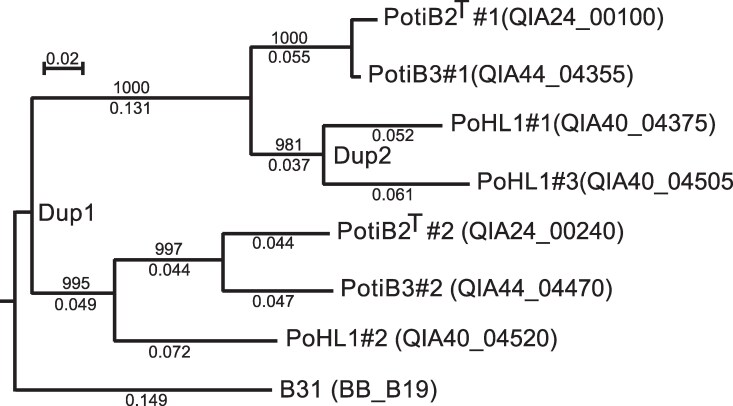
*Borrelia lusitaniae* OspC neighbor-joining tree. *B. lusitaniae* OspC protein amino acid sequences with *Borrelia burgdorferi* B31^T^ OspC for comparison were aligned, and a neighbor-joining tree was constructed by Clustal X (Larkin et al. 2007). The tree was rooted using B31 as the outgroup. Bootstrap values from 1,000 trials are shown above the major branches and fractional distances below the branches. A fractional distance scale bar is shown at the upper left. Isolate and OspC gene accession numbers are indicated at the right end of each branch. Two duplication events can be discerned. The first duplication event (labeled as “Dup1”) occurred in the common ancestor of all *B. lusitaniae* and the second duplication (labeled as “Dup2”) occurred only in the PoHL1 lineage. Each duplication event creates two sets of genes that are paralogous to each other, while genes within each numbered set are orthologous to each other.

The seven OspC proteins encoded by the three *B. lusitaniae* cp26s form two branches in the neighbor-joining tree of their amino acid sequences (Fig. 4). In one branch the PotiB2^T^#1, PotiB3#1, PoHL1#1 and PoHL1#3 OspC proteins are all >83% identical to one another, and in the second branch PotiB2^T^#2, PotiB3#2, and PoHL1#2 proteins are all >81% identical (see Fig. 3 for gene locations). Members of these two branches are <68% identical to one another. Two *ospC* duplication events can be discerned (Fig. 4). The first duplication associated with the cp26 dimerization in the common ancestor of these three genomes, resulted in two *ospC* genes. The second duplication of a smaller *ospC* gene-containing region occurred within the PoHL1 lineage, resulting in its three *ospC* genes. In both of these events it is not known whether a simple duplication was followed by *ospC* divergence or if two already different cp26s were participants. These three *B. lusitaniae* strains are the only BBSL isolates known to harbor multiple *ospC* genes, and it seems likely that this is a universal and important property of the *B. lusitaniae* species. It remains to be determined if the multiple *ospC* alleles are concomitantly expressed or alternatively expressed in different situations.

#### Cp32s

The BBSL cp32 plasmids are circular prophage DNAs (Eggers et al. 2001; Zhang and Marconi 2005; Wachter et al. 2023; Faith et al. 2024), and members of this plasmid family have been found in all previous carefully examined BBSL genomes except *B. garinii* Far04 and closely related isolates (Casjens et al. 2018; Margos et al. 2023). These plasmids are typically between 29 and 31 kbp long, but there are several cases of circular cp32 dimers about 60 kbp in length (for example in *B. burgdorferi* strains JD1, 94a and 118a (Casjens et al. 2012; Casjens et al. 2017; Margos et al. 2017) and *B. bavariensis* (Becker et al. 2020; Hepner et al. 2023)). These appear to be fusions of different cp32s formed by homologous recombination rather than a dimerization of a single plasmid. The circular cp32-28-4 plasmids in the genome sequence reported here have syntenic gene contents with the cp32 plasmid family, but they carry a lp28-4 type rather than a cp32 type PFam32 protein. The latter protein is about 6% different from its closest linear lp28-4 plasmid-encoded PFam32 homolog (that in *B. afzelii* strain PKo lp28-4), but it seems unlikely that this difference is sufficient to confer a different compatibility (Casjens et al. 2017; Casjens et al. 2018). The single PotiB2^T^ cp32 plasmid (cp32-12 + 28-4) is 58,174 bp long and is a head-to-tail dimer of typical cp32 monomer plasmids. The two parts encode PFam32 proteins that are closely related to those previously defined in cp32-12 and cp32-28-4 plasmid types. The PotiB3 sequence data assembled into three cp32 monomer circles (cp32-1, cp32-12 and cp32-28-4). PoHL1 contains only one cp32, a cp32-28-4 monomer. Previously, lp28-4 type PFam32 plasmid partition genes had been found only on linear plasmids, but we reported briefly that such lp28-4 type PFam32-carrying circular cp32s are also present in *B. japonica, B. sinica, B. tanukii, Borrelia valaisiana*, and *Borrelia yangtzensis* isolates (Akther et al. 2024). Thus, they are not unique to *B. lusitaniae*.

In addition to proteins thought to be required for growth as a bacteriophage, like cp32s from other BBSL species the *B. lusitaniae* cp32 plasmids encode “Erp” surface lipoproteins in their variable region 3 and “Mlp” and “Rev” proteins in their variable region 1 (Casjens et al. 2012; Brisson et al. 2013). These proteins form variable and complex sequence relationship groups that are reported to bind various host macromolecules including fibronectin, laminin, heparan and plasminogen, as well as affecting the host immune system by binding complement regulators factor H, several factor H-related proteins and complement factors C1s and C1r (reviewed by Stevenson and Brissette 2023).

#### Lp17

**Fig. 5.**
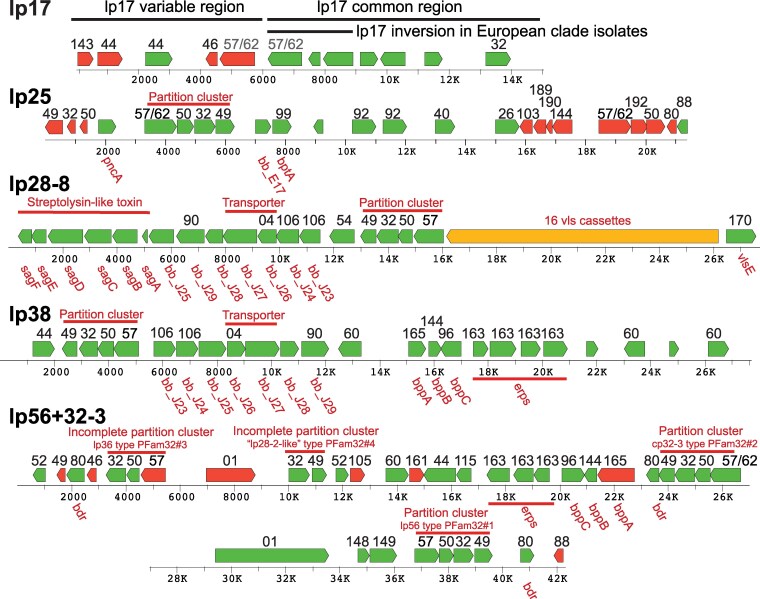
Gene maps of the five *Borrelia lusitaniae* PotiB2^T^ variable linear plasmids. Open reading frame maps are shown where predicted genes that appear to be intact are green, pseudogenes with broken reading frames are red, and *vls* cassettes are in orange. Black numbers above the genes indicate their Paralogous Protein Family (PFam) according to the definitions of Casjens et al. (2000, 2012). Red labels indicate functions and other names given to various genes. A base pair scale is shown below each map.

Linear lp17 plasmids are present in all previously sequenced BBSL genomes except one *B. carolinensis* isolate where it may have been lost in culture (Casjens et al. 2017; Casjens et al. 2018; Akther et al. 2024). The *B. burgdorferi* B31^T^ lp17 has been implicated in several aspects of mouse infection (Casselli et al. 2019; Wachter et al. 2023). Like other characterized lp17s, the PotiB2^T^ lp17's rightmost approximately 8 kbp “common region” is similar in sequence to the parallel regions of other BBSL lp17s (Casjens et al. 2017; Casjens et al. 2018). In the three *B. lusitaniae* lp17s this common region contains the ∼4 kbp inversion (relative to *B. burgdorferi*) that is also present in the lp17s of *B. garinii. B. afzelii, B. sinica, B. spielmanii, B. tanuki, B. turdi, B. valaisiana* and *B. yangtzensis* (Casjens et al. 2018; Akther et al. 2024), supporting the idea that this *B. lusitaniae* plasmid shares common evolutionary ancestry with the BBSL Eurasian clade. The left portion of lp17s varies among BBSL species and often varies among isolates of the same species. Figure 5 shows that the ∼6 kbp variable left-end portion of PotiB2^T^ lp17 contains several pseudogenes (of intact genes on linear plasmids in other BBSL isolates) and an apparently intact PFam44 lipoprotein gene of unknown function. The latter gene's closest relatives outside *B. lusitaniae* are 63–74% identical to those encoded by other plasmid types in *B. garinii, B. bavariensis, B. turdi, B. afzelii* and *B. spielmanii*. The *B. afzelii* and *B. spielmanii* PFam44 homologs, for example, reside on lp28-3 linear plasmids.

PotiB3 and PoHL1 lp17's are similar to that of PotiB2^T^ but each has organizational differences in the left terminal several kbp, which includes a second PFam44 gene in the PoHL1 plasmid. This is typical of the “variable” linear plasmids in BBSL species, where cognate plasmids in different isolates of the same species often have a few organizational differences but overall are considerably more like one another than they are like cognate plasmids from other species (Casjens et al. 2017; Casjens et al. 2018). Comparison of a 5 kbp section in the common region of lp17 that contains no major indels (bp 7001–12000, PotiB2^T^ coordinates) shows that PotiB2^T^ and PotiB3 are 99.2% identical in that region, whereas PoHL1 lp17 is 96.8 and 97.8% identical to the parallel PotiB2^T^ and PotiB3 regions, respectively. This again supports the separate lineage of PoHL1.

#### Lp25

The PotiB2^T^ linear lp25 plasmid, like lp25s from most other BBSL genomes, carries *pncA* and *bptA* genes as well as a homolog of *B. burgdorferi* B31^T^ gene *bb_e17* (Fig. 5). The *pncA* gene encodes a nicotinamidase that is essential for strain B31^T^ virulence in mice (Purser et al. 2003; Jewett et al. 2011), and *bptA* is required for persistence in ticks in strain B31^T^ (Revel et al. 2005). The PotiB2^T^, PotiB3 and PoHL1 lp25's are similar to one another; however, the PotiB3 plasmid has an approximately 5 kbp insertion relative to PotiB2^T^ near its right end, and PoHL1 lp25 has a several kbp replacement at its right end relative to the other two. Comparison of a 10 kbp section (bp 1-10,000, PotiB2^T^ coordinates) in their common left halves shows that this part of PotiB2^T^ lp25 is 98.9% identical to the parallel region of PotiB3 lp25, and these two are both 95.6% identical to this region of PoHL1 lp25.

#### Lp28-8

The three *B. lusitaniae* isolates carry very similar lp28-8 plasmids. These plasmids encode the following apparently important genes: (i) *vls* cassettes and *vlsE* expression locus, (ii) streptolysin S-like peptide toxin “*sagABCDEF*” synthesis genes, (iii) a PFam54 gene, and (iv) homologs of *B. burgdorferi* B31^T^ lp38 genes *bb_J23* through *bb_J29* (Fig. 5). The sixteen tandem *vls* cassettes are used to modify the *vlsE* expression locus (by homology with *B. burgdorferi* strains B31^T^ and JD1; see Zhang et al. 1997; Coutte et al. 2009; Verhey et al. 2019; Coburn et al. 2021; Norris and Brangulis 2024). As in other *vls/vlsE* loci, the G + C content of about 52% is much higher than the remainder of the genome, and there is a long, several hundred bp inverted repeat with intermediate G + C content between *vlsE* and the *vls* cassettes (see Casjens et al. 2012). As in other BBSL species, the rapidly evolving *vls* regions of the three *B. lusitaniae* lp28-8s are rather different in sequence despite having a conserved organization.

The *B. lusitaniae* lp28-8 *sagABCDEF* genes are similar to those carried by lp28-8 plasmids in *B. afzelii, B. japonica, B. spielmanii, B. turdi* and *B. valaisiana* (Molloy et al. 2015; Casjens et al. 2018; Akther et al. 2024). The *sagC* and *sagD* (also called *borC* and *borD*) genes were previously detected by polymerase chain reaction methods in the two *B. lusitaniae* isolates tested (PotiB2^T^ and PotiB3); however, even though *sagB* (*borB*) is present in the sequences reported here it was not detected by Molloy et al. (2015), presumably because the detection primers were not designed from *B. lusitaniae* sequence. The biological role of the toxin product of these genes is not known.

The specific role of the *B. burgdorferi* B31^T^  *bb_J23* through *bb_J29* gene cluster is also unknown, although two of the genes (*bb_J26* and *J27*) encode putative ABC-type transporter subunits (Casjens et al. 2000). This cluster is present on lp38 in B31^T^, and similar clusters are found across the BBSL species, often on lp28-8 plasmids as in the three *B. lusitaniae* isolates. They are also found on other plasmids such as lp17, lp28-3, lp28-4 and lp28-12 in other species (listed in Supplementary Table 2) (Casjens et al. 2012; Casjens et al. 2017; Casjens et al. 2018; Margos et al. 2019; Hepner et al. 2023; Margos et al. 2023; Akther et al. 2024). We note that this cluster is present in multiple copies in the following isolates: PotiB2^T^ (on lp28-8 and lp38), *B. turdi* Ya501 (on lp28-4 and lp28-12), and *B. valaisiana* VS116 and 89B13 (on lp28-8 and lp28-3), and *B. turdi* 047-3 (on lp28-8, lp28-4 and lp28-12). The common occurrence of the *J23-J29* cluster in BBSL isolates and its presence on plasmids in relapsing fever *Borrelia* species (for example, *B. anserina* Es plasmid lpJ (Elbir et al. 2017) and *B. hermsii* YBT plasmid contig0014 (accession No. CP005719)) suggests an importance for *Borrelia* bacteria that is not yet understood.

Highly syntenic lp28-8s that carry the *vls/vlsE*, *sagABCDEF* and *J23*-*J29* gene clusters are found exclusively in the Eurasian clade of the BBSL species (see Akther et al. (2024) for discussion of the Eurasian and North American clades). They are present in *B. afzelii* PKo, K78 and BO23, *B. japonica* HO14 and Miyazaki2E, *B. spielmanii* A14S and PMew, *B. turdi* 047-3, and *B. valaisiana* VS116, 89B13 and Am501 in addition to the three *B. lusitaniae* isolates (Supplementary Fig. 2 and Supplementary Table 2). North American clade species' lp28-8s typically carry a *vls/vlsE* region, but do not carry the other lp28-8 genes discussed here. Strikingly, the genes in the Eurasian type lp28-8 plasmid are unusually (for *Borrelia* linear plasmids) closely packed with little non-protein-coding sequence between them. Among the BBSL linear plasmids, only the lp54 plasmids have a similar close packing of genes. Again, the *sagABCDEF* and *J23-J29* portions of the PotiB2^T^ and PotiB3 lp28-8s are more similar to each other than they are to these regions in PoHL1 (analysis not shown).

#### Lp38

The PotiB2^T^ linear lp38 plasmid carries homologs of the *B. burgdorferi* B31^T^ lp38 genes *bb_J23* through *bb_J29* genes as discussed above, and unlike other known lp38s, it also carries an approximately 6 kbp region that contains four apparently cp32-derived *erp*-like lipoprotein genes (above) (Fig. 5). In addition, PotiB2^T^ lp38 encodes three PFam60 proteins. The detailed roles of PFam60 genes remain unclear, although *B. burgdorferi* PFam60 protein E31 is important in movement of the bacterium from the gut to the hemolymph during tick feeding (Zhang et al. 2011) and is a distant relative of the PFam54 proteins (see lp54 section below) (Brangulis et al. 2020). The genome sequences of PotiB3 and PoHL1 do not contain an lp38 plasmid.

#### Lp56 + 32-3

The complex PotiB2^T^ linear lp56 + 32-3 plasmid encodes four different PFam32 proteins that belong to the lp56, cp32-3, lp36 types and a previously unknown type. The latter (#4 in Supplementary Fig. 1) is about 24% different from its closest relatives, the lp28-2 type PFam32 proteins. This difference suggests that it represents a possibly unique compatibility type that has not been previously observed in other BBSL plasmids. We also note that the lp56-like PFam32 protein is about 16% different from its closest known relative, that of strain B31^T^ lp56 (#1 in Supplementary Fig. 1). This difference is sufficient to perhaps specify a novel compatibility type, but we assume here that it is the lp56 type. Experimental studies will be required to understand these compatibility issues (see Casjens et al. (2018) and Schwartz et al. (2021) for more detailed discussions of these questions). The lp56 and cp32-3 type PFam32 genes both lie in canonical four-gene “partition clusters” with PFam57/62, PFam50 and PFam49 genes (Casjens et al. 2000; Casjens et al. 2010; Casjens et al. 2012; Schwartz et al. 2021), whereas the other two are not in such clusters (Fig. 5). Thus, this plasmid was named lp56 + 32-3 after the two PFam32 gene types that are present in complete clusters. This plasmid is linear and its sequence is complete since both telomeric “wraparound” sequences were clearly present in the SMRT sequence data. Nonetheless, it carries ten genes typically found on *Borrelia* cp32 circular plasmids that include three *erp*-like lipoprotein genes. Finally, this PotiB2^T^ plasmid also carries an apparently intact PFam01 type restriction/modification gene (Kawabata et al. 2004; Rego et al. 2011) and several PFam80 *bdr* genes of unknown function (Zuckert et al. 1999). The genome sequences of PotiB3 or PoHL1 do not contain a homolog of this plasmid.

#### Lp54

The *B. lusitaniae* lp54s are largely syntenic to the lp54 plasmids present in other BBSL species; however, they have several unique organizational differences from lp54s in other BBSL species (Supplementary Fig. 3). The PotiB2^T^ and PotiB3 lp54s have four tandem PFam60 lipoprotein genes (above) at their left ends while PoHL1 has three such genes. The *B. garinii, B. afzelii, B. bavariensis, B. turdi* and *B. spielmanii* lp54s have single PFam52 and PFam60 genes in their left-end extensions, but the other 17 BBSL species have none (Casjens et al. 2017; Casjens et al. 2018; Akther et al. 2024). All three *B. lusitaniae* lp54s have four tandem copies of a homolog of *B. burgdorferi* B31^T^ lp54 gene *a36* and two tandem copies of *B. afzelii* PKo lp54 gene BafPKo_A0029 (the latter has no B31^T^ homolog). Also, unlike other known lp54s, the *B. lusitaniae* lp54s lack a right terminal *thyX* thymidylate synthase gene (Zhong et al. 2006); this is not due to sequence data failing to reach the linear plasmid end, because the SMRT sequence data has clear telomeric wraparound sequences at this location. We note that *B. japonica* lp54 is also missing *thyX*, but it has a very different right-end structure, and that *B. americana* and *B. turdi thyX* genes are present as pseudogenes (Akther et al. 2024). When the few indels >10 bp are removed, the constant region between bps 8,000 and 30,000 of PotiB2^T^ lp54 is 99.2% identical to that of PotiB3 and 96.2% identical to that of PoHL1.

Like other BBSL lp54s, the *B. lusitaniae* lp54s have tandem arrays of paralogous PFam54 genes near their right ends (Supplementary Fig. 3) (*B. japonica* arrays are truncated by their unusual lp54 structure). The genes in the central part of these arrays are the most variable (Wywial et al. 2009; Casjens et al. 2018; Akther et al. 2024), and the arrays in sequenced BBSL genomes have 2 to 7 such “variable” genes. The PotiB2^T^ and PotTiB3 arrays are very similar and have five apparently intact PFam54 genes in the “variable region”. Three of these encode PFam54 proteins that are ≤51% identical to homologs in other BBSL species. This region of the PoHL1 array has seven apparently intact genes of which five encode proteins that are ≤41% identical to those known in other species.

### Telomeres

**Fig. 6.**
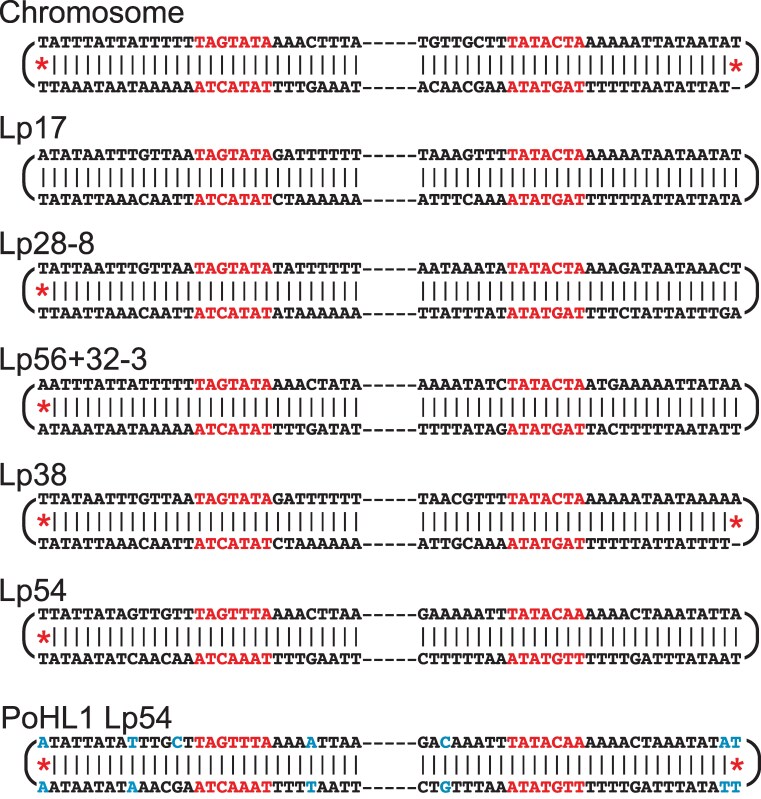
*Borrelia lusitaniae* linear replicon telomere structures. The top six replicons in the figure are the PotiB2^T^ linear chromosome and plasmids. Their top strands are 5′ to 3′ left to right and bottom strands are 3′ to 5′ left to right. Red text marks the most highly conserved sequence motif in the telomere region. For comparison, PoHL1 lp54 is shown at the bottom, where blue text indicates PoHL1 sequence that is different from PotiB2^T^. The DNA strand is continuous around the closed hairpin ends. Red asterisks mark telomere tips in which the terminal opposing bases are not complementary (see text).

SMRT sequencing runs read sequence continuously around *Borrelia*'s closed hairpin telomere tips and back along the opposite strand. These “wraparound” sequences show directly that, as has been reported for some plasmids in *B. burgdorferi* strains B31^T^ and CA-11_2A (Hinnebusch and Barbour 1991; Casjens et al. 1997; Fraser et al. 1997; Tourand et al. 2009; Faith et al. 2024), the PotiB2^T^ linear replicon DNA strands are continuous around the telomere tips. Complete telomere sequences at both ends were determined for the linear PotiB2^T^ chromosome and all linear plasmids except lp25. These twelve telomere sequences are shown in Fig. 6. The telomeric regions have exceptionally high A + T content and contain the same 5′-TAGTATA-3′ sequence motif (or a minor variation thereof) present in other Lyme agent *Borrelia* telomeres (Casjens et al. 1997). Previous chemical sequencing around three *B. burgdorferi* B31^T^ plasmid telomere tips found that they had complementary bases in the two strands all the way to their tips (Hinnebusch and Barbour 1991). This could be considered somewhat surprising, since the terminal bases are expected to be sterically prevented from physically pairing in a DNA hairpin. Thus, there is no known physical reason for them to be complementary. In agreement with this idea, only five of the twelve sequenced PotiB2^T^ telomeres have complementary bases in the two strands all the way to their tips. The imperfect complementarity at other seven tips is indicated by red asterisks in Fig. 6. The chromosome and lp38 right ends have a single “unpaired” base at the tip, while the left ends of the chromosome and plasmids lp28-8, lp56 + 32-3, lp38 and lp54 have noncomplementary bases at the terminal position of the two strands. Some of the PotiB3 and PoHL1 linear replicons also have terminal noncomplementary bases, but they are not always identical to those of PotiB2^T^ (see for example PoHL1 lp54 in Fig. 6). If replication proceeds through a head-to-head dimer circle as has been proposed (Casjens 1999; Picardeau et al. 1999), then these terminal noncomplementary bases should “flip-flop” between T-T and A-A at the chromosome left end, for example, in alternate rounds of replication (see Baroudy et al. 1982). Our analysis of telomeres in other BBSL species (Akther et al. 2024) shows that noncomplementary bases at telomere tips are also present in a number of those genomes (S. Casjens, unpublished). In addition, Faith et al. (2024) reported several telomere sequences in *B. burgdorferi* isolate CA-11_2A, and although the authors did not discuss it, seven of the 12 telomere sequences shown in Figure 10 of their study have noncomplementary terminal bases. Thus, terminal noncomplementary bases are not unique to *B. lusitaniae* and may be common in BBSL species. A more comprehensive analysis of *Borrelia* hairpin telomeric sequences will be presented in a subsequent publication.

## Summary

The *B. lusitaniae* chromosomes are very similar to, and syntenic with those of the other BBSL species, and single nucleotide variant analysis of their chromosomes indicates that they reside in the evolutionary branch that includes the other Eurasian BBSL species *B. afzelii, B. garinii, B bavariensis, B. sinica, B. yangtzensis, B. japonica, B. valaisiana, B. turdi, B. tanukii* and *B. spielmanii* (Akther et al. 2024). The analysis presented here shows that, overall, the *B. lusitaniae* plasmids conform largely to the patterns observed with other members of the BBSL group. Only one intact copy of any PFam32 compatibility gene type is present in any given isolate, lp17 plasmids are universally present and have especially variable left ends, lp25 carries the *pncA* and *bptA* genes found on most other lp25s, lp28-8 carries *sagABCDEF* toxin genes and a *vls/vlsE* region and is very similar to the lp28-8 plasmids in the Eurasian BBSL clade. The cp32 prophage plasmids are quite typical of such plasmids in BBSL species. Plasmids lp38 and lp56 + 32-3 appear to have been fairly recently rearranged relative to other species, and they carry homologs of genes typically found on the lp28 type and cp32 type plasmids in other species. The more variable linear plasmids (all those except lp54s, the right halves of lp17s, and some lp28-8s) in different BBSL species carry different overlapping sets of genes, and the organization of these genes varies among species, including *B. lusitaniae*, due to the apparently ongoing occurrence of rearrangement events. Such apparently random rearrangements can generate gene fragments, so it is not surprising that the *B. lusitaniae* variable linear plasmids, like those of other BBSL species, harbor a smattering of pseudogenes (Fig. 5).

An important unique aspect of the *B. lusitaniae* genomes is their dimeric cp26 plasmids. The *B. lusitaniae* cp26s are head-to-tail dimers of the canonical cp26 where in most cases one member of the original homologous gene pairs has suffered obviously debilitating mutations. Nonetheless, at least one member of each of the normally present cp26 gene types appears to be intact. This has resulted in two divergent, intact *ospC* genes in PotiB2^T^ and PotiB3 and three in PoHL1 (the latter due to a second shorter tandem duplication). These are the only known BBSL isolates that naturally carry multiple *ospC* genes. OspC is an important protein in mammalian infection (Grimm et al. 2004; Tilly et al. 2006), but it is not clear how more than one OspC type might be advantageous. It could be related to the unique ability of *B. lusitaniae* to utilize reptile as well as mammalian hosts. Other atypical features of the *B. lusitaniae* plasmids are the lack of a *thyX* gene and presence of three or four PFam60 genes on lp54.

The division of *B. lusitaniae* into two clades by MLST analysis—one from southern Portugal and North Africa that includes isolates PotiB2^T^ and PotiB3 and one from northern Portugal and central Europe that includes PoHL1—is strongly supported by the three genome sequences reported here. The sequences of the main chromosomes of isolates PotiB2^T^ and PotiB3 are more similar to each other than they are to PoHL1, and similar sequence relationships among the plasmids cp26, lp17, lp25, lp54, and the non-*vls/vlsE* portion of lp28-8 all fit very well with these two clades. The clear division of plasmid sequence types suggests that plasmid gene exchange between the two clades is not rapid. The genome sequences reported here will promote further study of *B. lusitaniae's* molecular lifestyle as well as serve as a foundation for further understanding of its natural population genetics and development of species- and clade-specific detection methods.

Finally, we discovered that the two DNA strands of *Borrelia* telomeres often have noncomplementary nucleotide bases at their tips. According to current models for replication of covalently closed hairpin telomeres, this should result in flip-flop of these noncomplementary bases in alternate replication rounds. Proof of such flip-flops is technically difficult but if obtained would provide support for the current replication model.

## Supplementary Material

jkaf319_Supplementary_Data

## Data Availability

The genome sequencing project is archived in the NCBI database under BioProject PRJNA431102 under BiosamplesSAMN10141377, SAMN34060369 and SAMN34060368. Fully assembled and annotated genome sequences are available in the NCBI nucleotide database; GenBank accession numbers for assembled replicons are given in Supplementary Table 1. An alignment and a tree of OspC sequences are available in the Github repository at https://github.com/weigangq/Bbsl_2023. Supplemental material available at G3 online.
