# Supplementary material for: Comparative genomics of Borrelia lusitaniae
Source: G3 (Bethesda). 2026 Jan 12;16(3):jkaf319. doi: 10.1093/g3journal/jkaf319 (PMC12958805; doi:10.1093/g3journal/jkaf319)
Supplement: jkaf319_Supplementary_Data [file jkaf319_supplementary_data.zip › Figure_S1_G3-2025-406463.pdf]

Figure S1.   PFam32 neighbor-joining tree

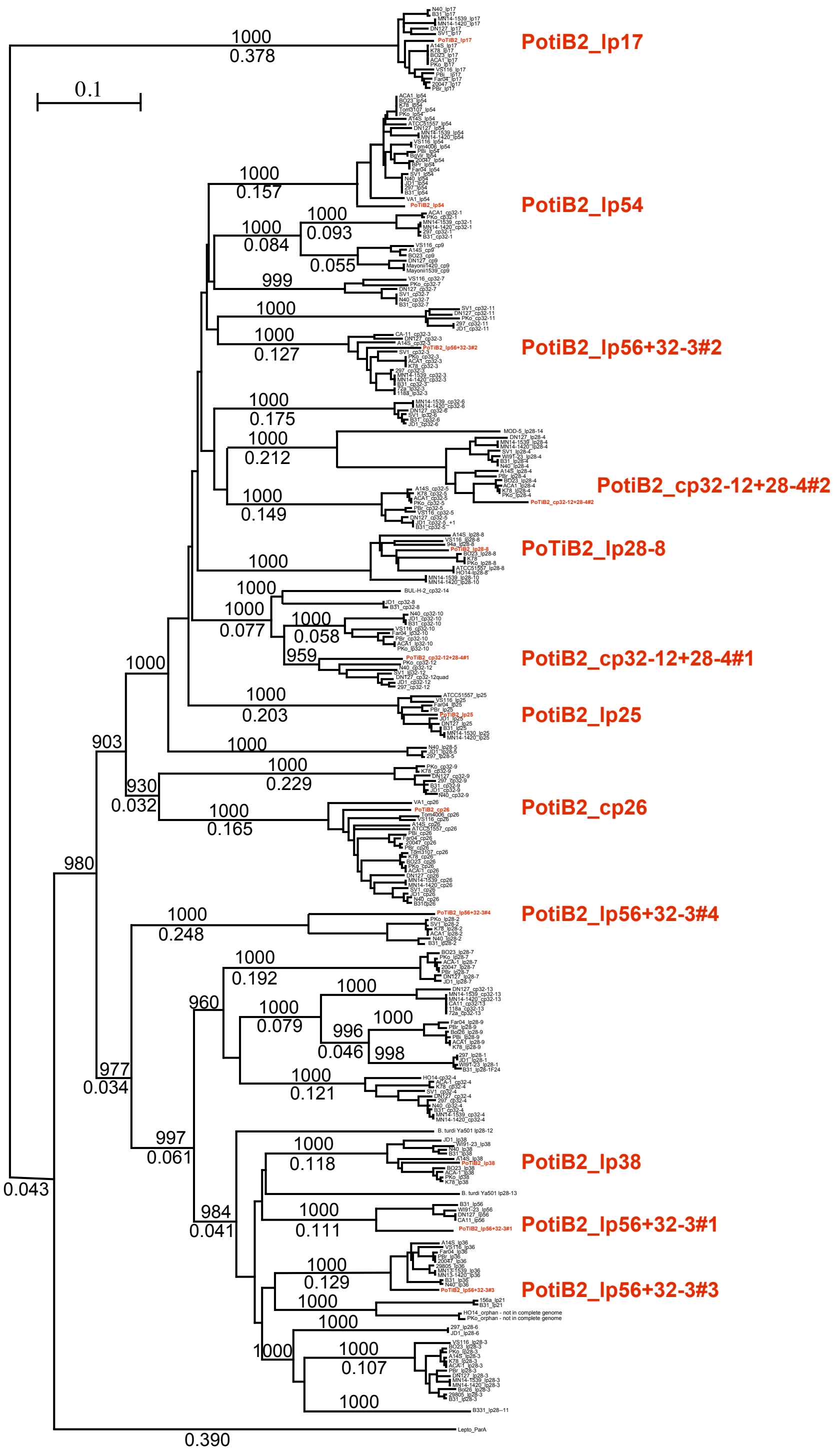

Figure S1. PFam32 neighbor-joining tree

The *B. lusitaniae* PFam32 protein and a sampling of previously reported PFam32 protein amino acid sequences were aligned and a neighbor-joining tree was constructed by Clustal X (Larkin *et al.*, 2007 Bioinformatics. 23:2947). Selected bootstrap values from 1000 trials are shown above the major branches and fractional distances below the branches. A fractional difference scale bar is shown at the upper left. The BBSL included representatives of all known BBSL PFam32 protein types were randomly chosen.

Strain names (small print) and plasmid names (large print) are indicated at the right of the branch tips. Lepto_ParA is the chromosomally encoded outgroup ParA protein from *Leptospira interrogans* strain UT126, a species in another spirochete genus.
