# Supplementary material for: Comparative genomics of Borrelia lusitaniae
Source: G3 (Bethesda). 2026 Jan 12;16(3):jkaf319. doi: 10.1093/g3journal/jkaf319 (PMC12958805; doi:10.1093/g3journal/jkaf319)
Supplement: jkaf319_Supplementary_Data [file jkaf319_supplementary_data.zip › Figure_S2_G3-2025-406463.pdf]

**SUPPLEMENTARY MATERIAL – Lopes de Carvalho *et al.***

Figure S2

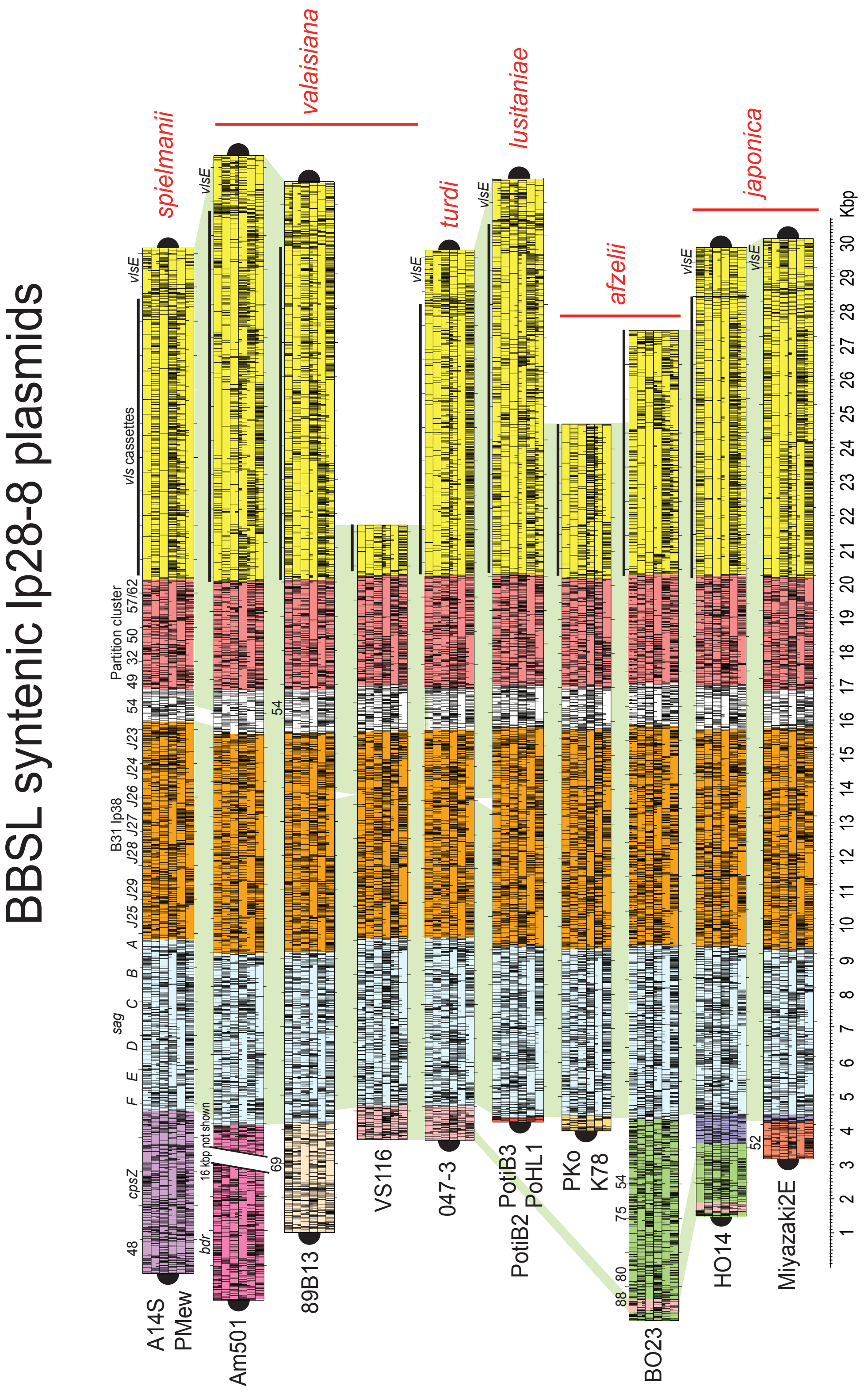

**Figure S2.** ***Borrelia lusitaniae*** **lp28-8 plasmids**

Linear plasmid reading frame maps are shown with isolate names on the left. On the right species names are shown in red text. The maps show the six possible reading frames (top three are rightward frames and bottom three leftward) with stop codons indicated by vertical lines that span the frame rectangle; potential start codons are indicated by short vertical lines (created with DNA Strider; Douglas, 1994 Methods Mol Biol. 25:181; see table S1 for accession numbers for sequences). Green shading indicates the homologous regions between adjacent plasmids. Gene names or protein (PFam) family names are given above the maps in italic or plan test, respectively. Black half circles indicate the ends in which the sequence includes the telomere (sequences lacking indicator this did not extend to the end of the plasmid). Yellow, rose, orange and light blue background colors on the maps mark the *vls*/*vlsE*, partition gene cluster, *bb_J23* - *bb_J29* cluster and *sagABCDEF* cluster, respectively. The left end differences, which are largely nonhomologous among the lp28-8s, are noted by other background colors.

The synteny among these plasmids is striking, although there are a few differences including degradation of the PFam54 gene in both *B. spielmanii* isolates and in *B. valaisiana* Am501, a frameshift in the Miyazaki2E PFam57/61 partition gene, partial truncation of the *bb_J26* homolog in VS116 and 047-3, an in-frame stop codon in047-3 *bb_J29*, and frameshifts in 047-3 *sagB* and *sagF*. It is not known if the frameshifts are real differences or sequencing errors.
