# Supplementary material for: Comparative genomics of Borrelia lusitaniae
Source: G3 (Bethesda). 2026 Jan 12;16(3):jkaf319. doi: 10.1093/g3journal/jkaf319 (PMC12958805; doi:10.1093/g3journal/jkaf319)
Supplement: jkaf319_Supplementary_Data [file jkaf319_supplementary_data.zip › Table_S1_G3-2025-406463.pdf]

SUPPLEMENTARY MATERIAL – Lopes de Carvalho *et al.*

Table S1. *B. lusitaniae* nucleotide sequence accession numbers

Isolate	Replicon	Accession	BioSample	BioProject
PotiB2^T	chromosome	CP124050	SAMN10141377	PRJNA431102
PotiB2^T	lp54	CP124049.	SAMN10141377	PRJNA431102
PotiB2^T	cp26	CP124047	SAMN10141377	PRJNA431102
PotiB2^T	lp17	CP124048	SAMN10141377	PRJNA431102
PotiB2^T	lp25	CP179533	SAMN10141377	PRJNA431102
PotiB2^T	lp28-8	CP179534	SAMN10141377	PRJNA431102
PotiB2^T	lp56+32-3	CP179535	SAMN10141377	PRJNA431102
PotiB2^T	lp38	CP179536	SAMN10141377	PRJNA431102
PotiB2^T	cp32-12+28-4	CP179537	SAMN10141377	PRJNA431102
PotiB3	chromosome	CP132468	SAMN34060368	PRJNA431102
PotiB3	lp54	CP132471	SAMN34060368	PRJNA431102
PotiB3	cp26	CP132469	SAMN34060368	PRJNA431102
PotiB3	lp17	CP132470	SAMN34060368	PRJNA431102
PotiB3	lp25	CP179538	SAMN34060368	PRJNA431102
PotiB3	lp28-8	CP179539	SAMN34060368	PRJNA431102
PotiB3	cp32-1	CP179540	SAMN34060368	PRJNA431102
PotiB3	cp32-12_	CP179541	SAMN34060368	PRJNA431102
PotiB3	cp32-28-4	CP179542	SAMN34060368	PRJNA431102
PoHL1	chromosome	CP132461	SAMN34060369	PRJNA431102
PoHL1	lp54	CP132464	SAMN34060369	PRJNA431102
PoHL1	cp26	CP132462	SAMN34060369	PRJNA431102
PoHL1	lp17	CP132463	SAMN34060369	PRJNA431102
PoHL1	lp25	CP179530	SAMN34060369	PRJNA431102
PoHL1	lp28-8	CP179531	SAMN34060369	PRJNA431102
PoHL1	cp32-28-4	CP179532	SAMN34060369	PRJNA431102
