# Supplementary material for: Comparative genomics of Borrelia lusitaniae
Source: G3 (Bethesda). 2026 Jan 12;16(3):jkaf319. doi: 10.1093/g3journal/jkaf319 (PMC12958805; doi:10.1093/g3journal/jkaf319)
Supplement: jkaf319_Supplementary_Data [file jkaf319_supplementary_data.zip › Table_S2_G3-2025-406463.pdf]

SUPPLEMENTARY MATERIAL – Lopes de Carvalho *et al.*

Table S2. BBSL isolates with full *bb_23 - bb_29* gene clusters

Table S2. Isolates with full *bb_23 - bb_29* gene clusters

Species	Isolate	Plasmid	Sequence reference or unpublished accession number
B. burgdorferi	B31, 64b, WI91-23, 29805, N40 , 297, 156a, 118a, CA-11_2A, 72a, 80a, NIH3, NIH5, NIH8, Sh-2-82 and others	lp38	Casjens et al. (1997; 2000; 2012), Schutzer et al. (2011), Akther et al. (2024)
B. americana	SCW-41, SCW-30h	lp38	CP179256; CP179253
B. afzelii	PKo, K78, BO23	lp28-8	Casjens et al. (2011), Schuler et al. (2015); CP018264
B. bavariensis	NT24	lp17	CP059019
B. garinii	PBr, Far04, 20047, NG-Z6, 17-29Z1, 17-54Z3, 17-56Z1, 17-58N4, 17-59N1, FNG-1Z1, FNG-2Z14	lp17	Casjens et al. (2011), Margos et al. (2023)
B. japonica	HO14, Miyazaki 2E	lp28-8	CP179486; CP179503
B. lusitaniae	PotiB2	lp28-8, lp38	CP179534; CP179536
	PotiB3, PoHL1	lp28-8	CP179539; CP179531
B. spielmainii	A14S, PMew	lp28-8	Schutzer et al. (2012); CP179556
B. turdi	Ya501	lp28-4, lp28-12	CP179626; CP179619
	047-3	lp28-8, lp28-4, lp28-12	CP179617; CP179609; CP179610
	TPT2017; T1990A, T2084	lp28-12†	Margos et al. (2019)
B. valaisiana	VS116, 89B13	lp28-8, lp28-3	Schutzer et al. (2012), Hepner et al. (2023)
	100B40	lp28-3	CP179628
	Am501	lp28-8	CP179635

† called lp30 in Acc. No. NZ_QBLM01000004.1, NZ_QBLN01000004.1,NZ_QBLO01000005.1

(see main text for references)
