# Supplementary material for: Comparative genomics of Borrelia lusitaniae
Source: G3 (Bethesda). 2026 Jan 12;16(3):jkaf319. doi: 10.1093/g3journal/jkaf319 (PMC12958805; doi:10.1093/g3journal/jkaf319)

SUPPLEMENTARY MATERIAL – Lopes de Carvalho *et al.*

Figure S3. *B. lusitaniae* lp54 gene map

An open reading frame map of *B. lusitaniae* lp54's is shown with green putative genes that appear to be intact; red genes are probable pseudogenes with broken reading frames. Black numbers above the genes indicate the Paralogous Protein Family (PFam) to which they belong (PFams are defined according to Casjens *et al.* (2000 Mol Microbiol 35:490 & 2012 PLoS One 7: e33280). Red labels above genes indicate some other names given to these genes. Black horizontal lines above mark duplications and quadruplications relative to other BBSL species that are present uniquely in the *B. lusitaniae* plasmids. A base pair scale is shown below the map.

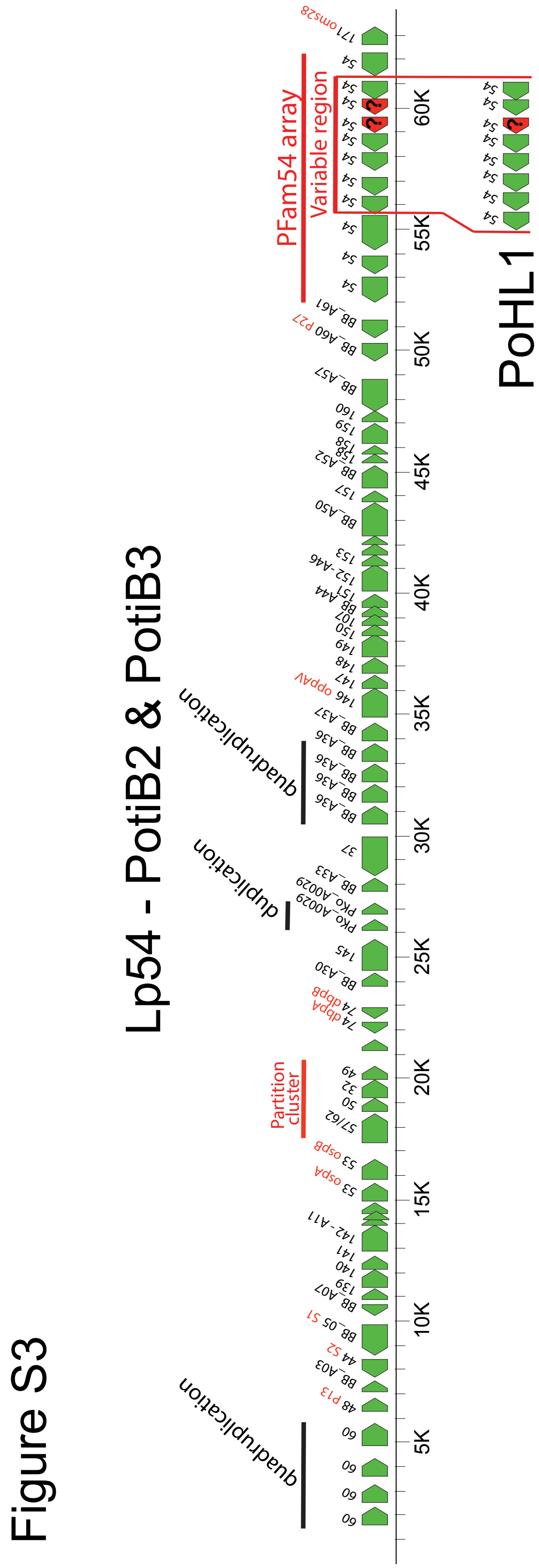

Supplement: jkaf319_Supplementary_Data [file Figure_S3_G3-2025-406463.pdf › Figure_S3_G3-2025-406463.pdf]
